# Routine Use of Prescription Adderall Leading to Non-cardiogenic Pulmonary Edema and Respiratory Failure

**DOI:** 10.7759/cureus.16371

**Published:** 2021-07-13

**Authors:** Alisha Khan, Bilal Talha, Vrinda Vyas, Usman Khan, Suman Rao, Amit Dhamoon

**Affiliations:** 1 Cardiology, State University of New York Upstate Medical University, Syracuse, USA; 2 Internal Medicine, State University of New York Upstate Medical University, Syracuse, USA

**Keywords:** adderall, non-cardiogenic pulmonary edema, respiratory failure, stimulant toxicity, side-effect of prescription adderall

## Abstract

A 47-year-old female with attention-deficit/hyperactivity disorder on prescription Adderall presented to the hospital with worsening dyspnea for the one-month duration. She was admitted to the medical intensive care unit with respiratory failure requiring non-invasive positive pressure ventilation. Cardiac catheterization confirmed the diagnosis of non-cardiogenic pulmonary edema. With the discontinuation of Adderall, use of BiPAP, and aggressive diuresis with loop diuretics, there was evidence of symptomatic, laboratory, and radiological improvement. Her symptoms were attributed to Adderall use as a diagnosis of exclusion. To our knowledge, this paper reports the first case of Adderall-induced non-cardiogenic pulmonary edema leading to respiratory failure. Although case reports of abuse or overdose of other stimulants such as amphetamine and cocaine leading to a plethora of cardiac and pulmonary complications such as acute respiratory distress syndrome (ARDS), cardiogenic pulmonary edema, and non-cardiogenic pulmonary edema exist, there are no reports that using Adderall at routine prescription doses can lead to these problems.

## Introduction

Our case presentation is the first case of Adderall-induced non-cardiogenic pulmonary edema leading to respiratory failure in a human. Although case reports of abuse or overdose of other stimulants such as amphetamine and cocaine leading to a plethora of cardiac and pulmonary complications such as acute respiratory distress syndrome (ARDS), cardiogenic pulmonary edema and non-cardiogenic pulmonary edema exist, there are no reports that using Adderall at routine prescription doses can lead to these problems. With the growing use of this medication, rare side effects are gradually coming to light.

## Case presentation

We provided care for a 47-year-old female with a past medical history of attention-deficit/hyperactivity disorder (ADHD), hypertension, and fibromyalgia who presented with worsening shortness of breath and difficulty breathing for the one-month duration. Her shortness of breath had begun suddenly while she was sitting and was not associated with pain. Her home medications included lisinopril 5 mg by mouth daily and Adderall 30 mg by mouth daily, which she had recently started taking again after being off of it for approximately four months. She had not required Adderall during that time as she was not working. She denied the use of illicit drugs and she claimed to have been using Adderall as prescribed by her psychiatrist.

In the Emergency Department, she was noted to be hypoxemic with blood oxygen saturation (SpO_2_) level around 50%, tachypneic with a respiratory rate of 49 breaths per minute, and tachycardic with a heart rate of 110 beats per minute. On 15 L O_2_/min via a non-rebreather mask, she was able to maintain a SpO_2_ in the low 90s. The initial pro-BNP was 5,486 pg/mL with EKG showing sinus tachycardia. Her urine toxicology resulted positive for amphetamines in the urine.

**Figure 1:**
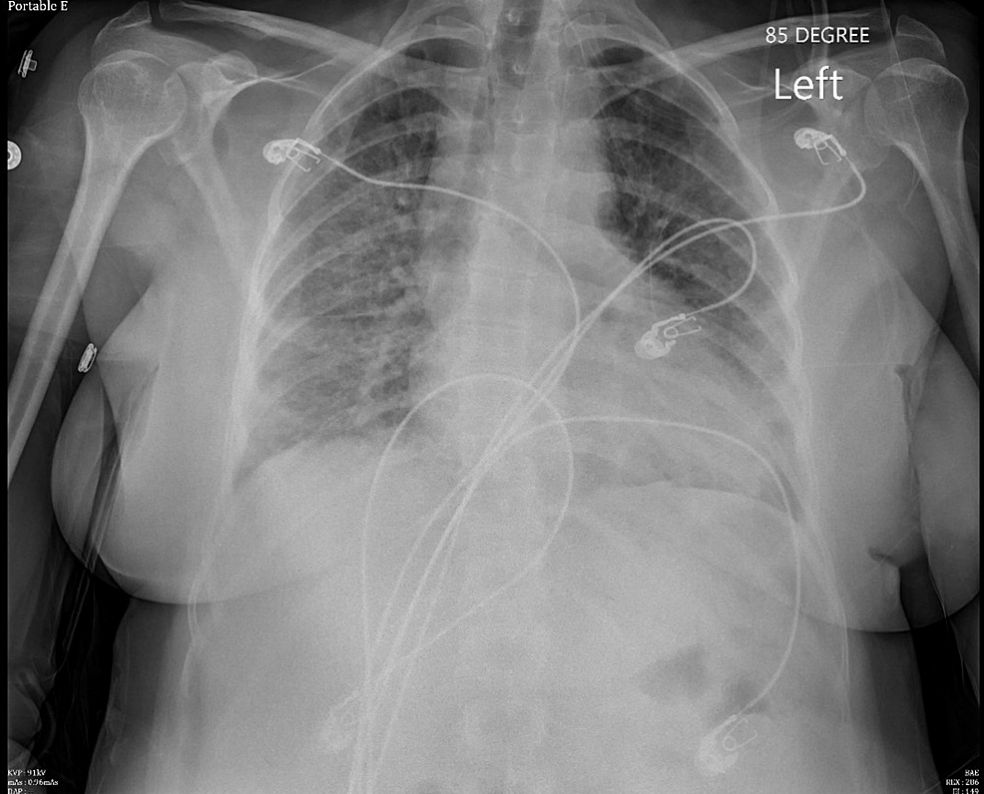
Chest x-ray on admission

Chest x-ray revealed prominent pulmonary vascular markings with scattered ill-defined opacities and associated interstitial thickening favoring underlying pulmonary edema and small bilateral pleural effusions (Figure 1).

**Figure 2:**
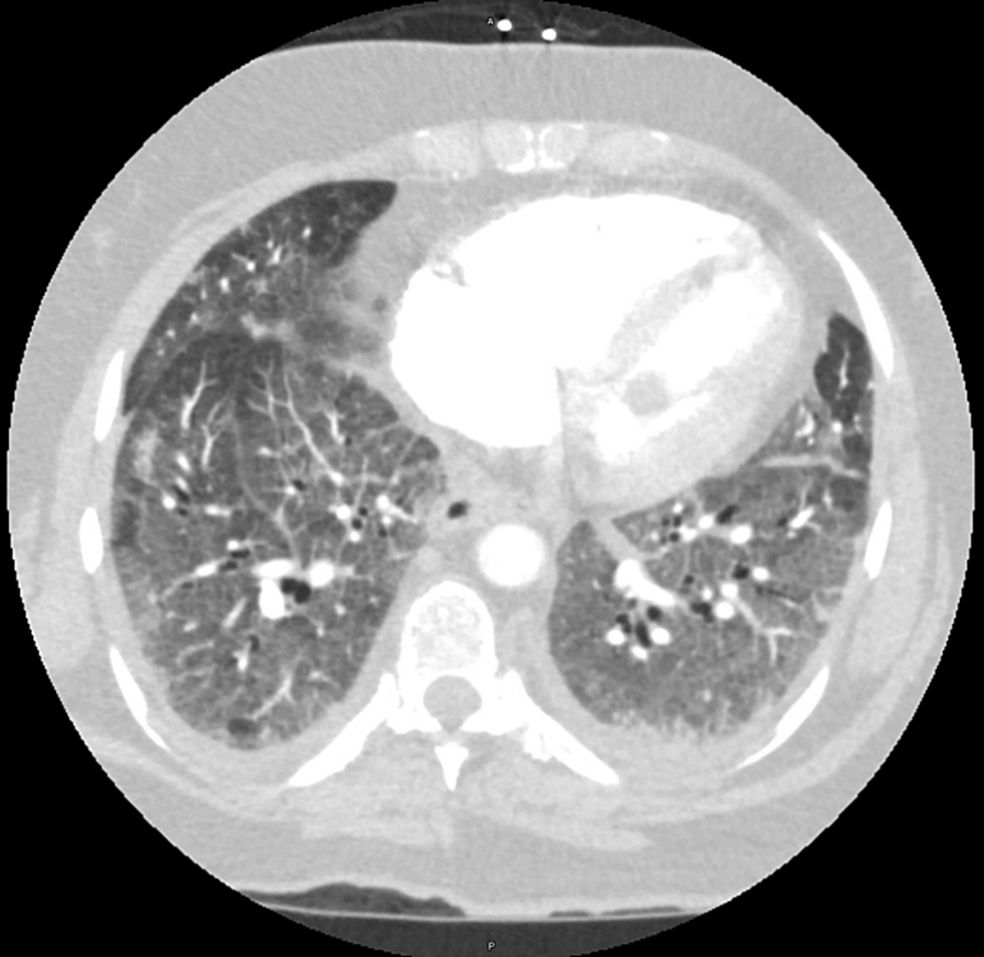
Computed tomography angiogram (CT-A) of the thorax on admission

CT angiogram of the thorax was negative for a pulmonary embolism; however, it showed bilateral diffuse ground-glass opacities with mild interstitial thickening, cardiomegaly with dilation of the right cardiac chamber, leftward interatrial septal bowing, and mild interventricular septal flattening, concerning for elevated right heart pressures (Figure 2).

She was admitted to the medical intensive care unit for acute hypoxemic respiratory failure where she was switched to bilevel positive airway pressure (BiPAP) for persistent respiratory distress. Her BiPAP settings consisted of inspiratory positive airway pressure (IPAP) of 14 cmH_2_O, expiratory positive airway pressure (EPAP) of 5 cmH_2_O and a fraction of inspired oxygen (FiO_2_) of 40%. Cardiology was consulted and a transthoracic echocardiogram was performed. Transthoracic echocardiogram revealed a normal ejection fraction of 55%-60% without diastolic dysfunction, an enlarged right ventricle with normal right ventricular systolic function, an estimated pulmonary arterial pressure of 50.96 mmHg and moderate tricuspid regurgitation.

She was placed on a furosemide drip, which improved her respiratory distress. Right and left heart catheterization was performed to confirm the pulmonary artery pressure and pulmonary capillary wedge pressure. Interestingly, it showed a normal pulmonary artery pressure of 33/15 mmHg (mean 22 mmHg) and a normal wedge pressure of 10/9 mmHg (mean 8 mmHg), confirming non-cardiogenic pulmonary edema. Of note, right ventricular pressure was 33/6 mmHg (mean 11 mmHg), right atrium pressure was 11/10 mmHg (mean 8 mmHg) and aortic pressure was 110/73 mmHg (mean 91 mmHg). In other words, right heart catheterization demonstrated that her pulmonary edema is unlikely to be from a cardiac etiology. A left heart catheterization revealed insignificant coronary artery disease. The pulmonary service was therefore consulted to aid us in determining the cause of her non-cardiogenic pulmonary edema.

Her extractable nuclear antigen panel resulted negative, other than a mildly elevated rheumatoid factor level of 31 IU/mL, a C-reactive protein level of 282 mg/L, and an erythrocyte sedimentation rate of 88 mm/hr. Infective workup including blood cultures, sputum cultures, white blood cell count, and respiratory viral panel resulted negative. HIV, thyroid function panel, and hepatitis C tests resulted negative. She presented to the hospital several months prior to the first documented case of COVID-19; therefore, she was not tested for it at the time.

After excluding all other causes, Adderall was deemed to be the culprit behind our patient’s non-cardiogenic pulmonary edema. We continued to diurese the patient with intravenous furosemide injections. With diuresis, her pro-BNP trended down from 5,486 to 32 pg/mL and the patient improved both clinically and radiographically.

Our patient was discharged home in a stable condition on room air with instructions to discontinue Adderall and to avoid any other stimulants. She was instructed to follow up with her primary care physician and psychiatrist for the management of her ADHD.

## Discussion

Adderall is a central nervous system stimulant containing amphetamines, which are used to manage ADHD. Specifically, Adderall is a 3:1 mixture of d- and l-enantiomers of amphetamine salts [1]. Amphetamines primarily increase the release of dopamine and norepinephrine into the synapse and, secondarily, decrease the reuptake of these neurotransmitters [2]. Norepinephrine-induced alpha-adrenergic stimulation results in vasoconstriction and increased total peripheral resistance [3]. Norepinephrine-induced beta-adrenergic stimulation results in increased heart rate, stroke volume, and blood flow to the skeletal muscles [3]. As a result, Adderall toxicity manifests itself most commonly as tachycardia, tachypnea, hyperactivity, mydriasis, tremors, and hyperthermia.

Here, we report a case of a 47-year-old female with ADHD who presented with worsening dyspnea, which was attributed to Adderall use as a diagnosis of exclusion. With the discontinuation of her medication, use of BiPAP, and aggressive diuresis with loop diuretics, there was evidence of symptomatic, laboratory, and radiological improvement.

Upon review of the literature, there are no human case reports of prescription Adderall-induced non-cardiogenic pulmonary edema. Although case reports of abuse or overdose of other stimulants such as amphetamine and cocaine leading to a plethora of cardiac and pulmonary complications such as ARDS, cardiogenic pulmonary edema, and non-cardiogenic pulmonary edema exist, there are no reports that using Adderall at routine prescription doses can lead to these problems [4,5].

Cardiogenic pulmonary edema from stimulant abuse is thought to result from acute and chronic cardiomyopathy, myocardial ischemia, hypertensive crisis, diastolic dysfunction, and arrhythmias [6]. On the other hand, non-cardiogenic pulmonary edema from stimulant abuse is postulated to result from alveolar epithelial and endothelial damage and occasionally sustained seizure activity [6]. However, as aforementioned, our patient strictly denied Adderall abuse and maintained that she was using her medication as prescribed.

Cocaine, another stimulant, is a known cause of both cardiogenic and non-cardiogenic pulmonary edema whether used intravenously or inhaled via smoking [4,7,8]. Pulmonary edema has been demonstrated in 77% to 85% of cocaine-related deaths in autopsy series [4,9].

ARDS is a clinical entity that requires the presence of non-cardiogenic pulmonary edema for diagnosis. Our patient was unlikely to have ARDS because her symptoms progressed gradually over one month, as opposed to the acute nature of ARDS. According to the Berlin ARDS definition, respiratory distress develops rapidly within one week of a known clinical insult [10].

## Conclusions

In conclusion, stimulants such as amphetamines can cause both cardiogenic and non-cardiogenic pulmonary edema. Drug-related lung injury is a rare event and is believed to be idiosyncratic. This lung injury cannot be attributed to the dose or duration of the drug. The diagnosis can become tricky when the patient is only on a prescription stimulant like Adderall and there is no history of illicit stimulant use as in the case presented above, as other causes of acute pulmonary edema need to be excluded first.
